# Response: Commentary: Correlation between Patent Foramen Ovale, Cerebral “Lesions” and Neuropsychometric Testing in Experienced Sports Divers: Does Diving Damage the Brain?

**DOI:** 10.3389/fpsyg.2016.01586

**Published:** 2016-10-24

**Authors:** Costantino Balestra, Peter Germonpré

**Affiliations:** ^1^Environmental and Occupational (Integrative) Physiology, Haute Ecole Paul Henri SpaakBrussels, Belgium; ^2^Research Division, Divers Alert Network EuropeBrussels, Belgium; ^3^Faculté des Sciences de la Motricité, Université Libre de BruxellesBrussels, Belgium; ^4^Center for Hyperbaric Oxygen Therapy, Military Hospital Queen AstridBrussels, Belgium

**Keywords:** diving, long-term effects, persistent foramen ovale, brain MRI, cerebral embolism

We are grateful to Dr. Gempp to let us clarify and explain our choices.

We would like to confirm that the quoted reference was the one that we have chosen. We indeed are aware of the other reference (Gempp et al., 2010), but we deliberately didn't choose this paper for reasons that are expressed by Dr Gempp himself: ≪.…we clearly showed […] in 34 military divers who strictly adhere to decompression procedures……≫. Since the decompression procedures that the French Navy are using are quite different of those used by the divers in our study (the speed of ascent, for instance, is roughly 33% faster in the French Navy procedures) and Navy divers strictly adhere to these procedures, as opposed to the more liberal approach (in both directions of conservatism) of recreational divers, this makes the two diver populations possibly very different. We are in fact aiming our work on recreational divers.

Considering the other references referring to white matter hyperintense spots, these MRI findings are actually still considered asymptomatic and their clinical significance is unknown; furthermore, although proposed in the literature, the morphology of these hyperintensities does not seem to indicate a definite vascular origin (Balestra et al., 2004).

In our Discussion, we have taken the time to explain some of the limitations of the studies that can be found in the literature. We furthermore encourage the reader to consider a Letter to the Editor which we wrote in reaction to one of the most cited articles on the topic (Torti et al., 2004), highlighting, among other methodological issues, the high risk of self-selection bias in such studies (Germonpre and Balestra, 2004).

As Dr Gempp adequately pointed out, the number of asymptomatic hyperintense white matter spots is lower in our study. This merely illustrates the importance of mitigating, when designing such studies, common biases like self-selection, specific (non-recreational) decompression procedures, non-recreational diver populations, non-randomization as well as non-homogenous samples. All these factors may result in a higher prevalence of asymptomatic white matter spots found by MRI imaging than are actually present in the actual intended study population.

We would like to thank Dr Gempp for his encouragement to move toward a prospective study in order to analyze adequately the relative risk of Patency of Foramen Ovale and decompression sickness in recreational SCUBA divers. We can assure him that such studies are well underway and will be published shortly.

## Author contributions

We answered to the commentary on our article after mutual acceptance of the manuscript proposed.

### Conflict of interest statement

The handling Editor declared a shared affiliation, though no other collaboration, with one of the authors CB and states that the process nevertheless met the standards of a fair and objective review. The other authors declare that the research was conducted in the absence of any commercial or financial relationships that could be construed as a potential conflict of interest.
